# Commensal *Enterobacteriaceae* as reservoirs of extended-spectrum beta-lactamases, integrons, and *sul* genes in Portugal

**DOI:** 10.3389/fmicb.2013.00080

**Published:** 2013-04-08

**Authors:** Elisabete Machado, Teresa M. Coque, Rafael Cantón, João C. Sousa, Luísa Peixe

**Affiliations:** ^1^REQUIMTE, Laboratório de Microbiologia, Faculdade de Farmácia, Universidade do PortoPorto, Portugal; ^2^CEBIMED, Faculdade de Ciências da Saúde, Universidade Fernando PessoaPorto, Portugal; ^3^Servicio de Microbiología, Hospital Universitario Ramón y Cajal, Instituto Ramón y Cajal de Investigación SanitariaMadrid, Spain

**Keywords:** ESBLs, CTX-M-14, TEM-153, class 1 and class 2 integrons, healthy volunteers

## Abstract

Bacteria colonizing the human intestine have a relevant role in the spread of antimicrobial resistance. We investigated the faecal carriage of extended-spectrum beta-lactamase (ESBL)-producing *Enterobacteriaceae* in healthy humans from Portugal and analyzed the distribution of *sul* genes and class 1 and 2 integrons. Faecal samples (*n* = 113) were recovered from healthy persons (North/Centre of Portugal, 2001–2004) and plated on MacConkey agar with and without ceftazidime (1 mg/L) or cefotaxime (1 mg/L). Isolates representing different morphotypes/plate and antibiotic susceptibility patterns (*n* = 201) were selected. Isolates resistant to sulfonamides and/or streptomycin, gentamicin, and trimethoprim were screened (PCR and sequencing) for *sul* genes (*sul1, sul2, sul3*) and class 1 and 2 integrons. Presence of ESBLs was inferred using the double disk synergy test (DDST) and further confirmed by PCR and sequencing. ESBL producers were selected for clonal analysis, plasmid characterization and conjugation assays by standard methods. ESBL-producing isolates were found in 1.8% (2/113) of samples, corresponding to *Escherichia coli* of phylogroups A (*n* = 1) and B1 (*n* = 1) carrying transferable *bla*_CTX-M-14_ and the new *bla*_TEM-153_, respectively. A 80kb IncK plasmid bearing *bla*_CTX-M-14_ was found, being highly related to that widely spread among CTX-M-14 producers of humans and animals from Portugal and other European countries. *sul* genes were found in 88% (22/25; *sul2*-60%, *sul1*-48%, *sul3-*4%) of the sulfonamide resistant isolates. Class 1 integrons were more frequently found than class 2 (7%, 14/201 *vs.* 3%, 6/201). Interestingly, gene cassette arrangements within these platforms were identical to those commonly observed among *Enterobacteriaceae* from Portuguese food-producing animals, although *aadA13* is here firstly described in *Morganella morganii*. These results reinforce the relevance of human commensal flora as reservoir of clinically relevant antibiotic resistance genes including *bla*_ESBLs_, and highly transferable genetic platforms as IncK epidemic plasmids.

## Introduction

Antimicrobial resistance has become a global public health problem, compromising the treatment of several infectious diseases. Acquisition of integrons and beta-lactamase genes by *Enterobacteriaceae* is increasingly recognized, being associated with resistance to multiple antibiotics (Machado et al., 2007; Coque et al., 2008; Bush, 2010; Pitout, 2010). The production of extended-spectrum beta-lactamases (ESBLs) constitutes one of the currently most spread and relevant antibiotic resistance mechanisms, compromising the use of several beta-lactams. Portugal is one of the European countries with higher rates of ESBL producers in the clinical setting, with a shift from TEM or SHV variants to CTX-M-types noticed since 2003 (Machado et al., 2007; ECDC, 2011).

Colonization of healthy subjects with antibiotic resistant *Enterobacteriaceae* could contribute to the amplification of resistant bacteria both at community and nosocomial settings (Rodríguez-Baño et al., 2008; Valverde et al., 2008). High rates of antibiotic resistant *Escherichia coli* fecal isolates of healthy humans have been reported in different countries (London et al., 1994; van de Mortel et al., 1998; Zhang et al., 1998; Sáenz et al., 2001; Briñas et al., 2002; Bruinsma et al., 2003; Nys et al., 2004; Bailey et al., 2010). In Portugal, spread of multidrug resistant bacteria, including ESBL producers, in hospitals, healthy food-producing animals, food products and aquatic settings has been described (Machado et al., 2007, 2008, 2009; Mendonça et al., 2007). Nevertheless, their occurrence and diversity in healthy populations is unknown. In order to better understand the epidemiology of multidrug resistant *Enterobacteriaceae* in Portugal and the contribution of different settings to the burden of infections with antibiotic resistant bacteria, we investigated, during the initial spread period of CTX-M enzymes, the faecal carriage of ESBL-producing *Enterobacteriaceae*, class 1 and class 2 integrons, and *sul* genes in Portuguese healthy humans.

## Materials and methods

### Bacterial isolates

From January 2001 to February 2004, a total of 113 nonduplicate faecal samples were recovered from randomly selected healthy humans [53% females; age ranging from 5 to 59 (*n* = 99) and 60 to 76 (*n* = 14) years old] living in the North and Centre of Portugal. Samples were from persons without previous exposure to antibiotic therapy, hospitals, or long-term care facilities at least in the 3-month period before sampling. Rectal swabs were immersed in transport medium, faeces suspended in 1 mL of saline, and aliquots of 200 μL seeded in MacConkey agar with and without ceftazidime (1 mg/L) or cefotaxime (1 mg/L). Presumptive *Enterobacteriaceae* (oxidase-negative facultative aerobic Gram negative rods) were selected for further studies. *Enterobacteriaceae* isolates recovered in the same time period from Portuguese hospitals (*n* = 4; 2003–2004) or marine coastal waters (*n* = 1; 2003) close to clandestine discharge points of water streams contaminated with faecal coliforms, and producing ESBL-types similar to the ones identified in this study, were also included for clonal investigation and/or plasmid relationships analysis (Machado et al., 2007, 2009).

### ESBL detection and antimicrobial susceptibility

Each different morphotype growing on MacConkey agar with ceftazidime or cefotaxime was screened for ESBL production by the double disk synergy test (DDST) (Jarlier et al., 1988), and susceptibility testing to non-beta-lactam antibiotics (aminoglycosides, quinolones, sulfonamides, trimethoprim, tetracyclines, chloramphenicol) was carried out in positive isolates using the standard disk diffusion method (CLSI, 2007). Morphotypes corresponding to non-ESBL producers recovered from MacConkey agar with and without antibiotics were tested to streptomycin, gentamicin, trimethoprim and sulfonamides (CLSI, 2007).

### ESBL characterization and epidemiological features

Characterization of ESBLs was performed by amplification of *bla* genes and sequencing (Table 1), and ESBL-producing isolates were further identified by API ID 32GN (bioMérieux, Marcy l'Étoile, France). Clonal relatedness of ESBL producers was investigated by pulsed-field gel electrophoresis (PFGE), randomly amplified polymorphic DNA (RAPD) (Machado et al., 2005, 2008), and multilocus sequence typing (MLST) (http://mlst.ucc.ie/mlst/dbs/Ecoli). *E. coli* phylogenetic groups were identified by a multiplex PCR (Clermont et al., 2000).

**Table 1:** **Primers used in this study**.

**Primer**	**Oligonucleotide sequence (5′–3′)**	**Gene**	**Reference**
TEM-F	ATG AGT ATT CAA CAT TTC CG	*bla*_TEM_	Rasheed et al., 1997
TEM-R	CTG ACA GTT ACC AAT GCT TA		
SHV-1	GGG TTA TTC TTA TTT GTC GC	*bla*_SHV_	Rasheed et al., 1997
SHV-2	TTA GCG TTG CCA GTG CTC		
CTX-M-F′	TTT GCG ATG TGC AGT ACC AGT AA	*bla*_CTX-M_	Edelstein et al., 2003
CTX-M-R′	CGA TAT CGT TGG TGG TGC CAT A		
CTXM1-F3	GAC GAT GTC ACT GGC TGA GC	*bla*_CTX-M_ (group I)	Pitout et al., 2004
CTXM1-R2	AGC CGC CGA CGC TAA TAC A		
Toho1-F2	GCG ACC TGG TTA ACT ACA ATC C	*bla*_CTX-M_ (group II)	Pitout et al., 2004
Toho1-1R	CGG TAG TAT TGC CCT TAA GCC		
CTXM825F	CGC TTT GCC ATG TGC AGC ACC	*bla*_CTX-M_ (group III)	Pitout et al., 2004
CTXM825R	GCT CAG TAC GAT CGA GCC		
CTXM924F	GCT GGA GAA AAG CAG CGG AG	*bla*_CTX-M_ (group IV)	Pitout et al., 2004
CTXM924R	GTA AGC TGA CGC AAC GTC TG		
CTX-M-9-F	GTG ACA AAG AGA GTG CAA CGG	*bla*_CTX-M_ (group IV, sequencing)	Simarro et al., 2000
CTX-M-9-D	ATG ATT CTC GCC GCT GAA GCC		
IntI1-F	GGT CAA GGA TCT GGA TTT CG	*intI1*	Mazel et al., 2000
IntI1-R	ACA TGC GTG TAA ATC ATC GTC		
5′CS	GGC ATC CAA GCA GCA AG	Class 1 integron variable region	Levesque et al., 1995
3′CS	AAG CAG ACT TGA CCT GA		
IntI2-F	CAC GGA TAT GCG ACA AAA AGG T	*intI2*	Mazel et al., 2000
IntI2-R	GTA GCA AAC GAG TGA CGA AAT G		
attI2-F	GAC GGC ATG CAC GAT TTG TA	Class 2 integron variable region	Machado et al., 2005
orfX-R	GAT GCC ATC GCA AGT ACG AG		
Sul 1-F	CGGCGTGGGCTACCTGAACG	*sul1*	ller and Espersen, Kerrn et al.
Sul 1-B	GCCGATCGCGTGAAGTTCCG		
Sul 2-F	GCGCTCAAGGCAGATGGCATT	*sul2*	ller and Espersen, Kerrn et al.
Sul 2-B	GCGTTTGATACCGGCACCCGT		
sul3F	GAGCAAGATTTTTGGAATCG	*sul3*	Perreten and Boerlin, 2003
sul3R	CATCTGCAGCTAACCTAGGGCTTTGGA		

The transferability of ESBL genes was assessed by filter mating assays with *E. coli* BM21R (nalidixic acid- and rifampicin resistant, lactose fermentation positive and plasmid-free) (Machado et al., 2008), and ESBL-encoding plasmids were identified by PCR-based replicon typing and further hybridization (*bla*_ESBL_, *rep* probes), as previously described (Novais et al., 2010). Plasmid relationships were established by comparison of restriction fragment length polymorphism (RFLP) patterns obtained after digestion with *Eco*RI, *Pst*I, and *Hpa*I restriction enzymes (Valverde et al., 2009).

### Characterization of integrons and *sul* genes

Isolates representing different morphotypes and resistance patterns to streptomycin, gentamicin, trimethoprim and/or sulfonamides were selected for screening of class 1 and class 2 integrons by PCR (Table 1), as these phenotypes are commonly associated with these genetic structures (Machado et al., 2005). Class 1 and class 2 integrons were further characterized by RFLP-typing and sequencing, as described (Machado et al., 2005). Integrons were designated by roman numbers and a subindex indicates the class to which each integron belongs, as previously described (Machado et al., 2005). Isolates resistant to sulfonamides were also screened for the presence of sulfonamide resistance genes (*sul1*, *sul2*, *sul3*) by PCR (Table 1).

## Results

### Epidemiological background

A total of 201 *Enterobacteriaceae* isolates representing different colony morphotypes and antibiotic susceptibility patterns were obtained. Resistance to at least one antibiotic was observed in 79 isolates, and the resistance rates were higher for streptomycin (36%, 73/201) than for trimethoprim (15%, 31/201), sulfonamides (12%, 25/201), or gentamicin (4%, 9/201).

The proportion of faecal carriage of ESBL-producing *Enterobacteriaceae* was 1.8% (2 of 113 samples), corresponding to samples recovered in 2003 from two females, aged 21 and 76 years, respectively.

### ESBL characterization

We identified *Escherichia coli* isolates producing CTX-M-14 (phylogroup A, ST665) or TEM-153 (phylogroup B1), a novel TEM-type enzyme differing from TEM-1 by three amino acid changes (E104K, M182T, and G267V) (GenBank accession number KC149518). TEM-1 enzyme was also identified in the CTX-M-14 producer. The CTX-M-14-producing *E. coli* isolate showed a phenotype of resistance against streptomycin, sulfonamides, trimethoprim, tetracyclines, chloramphenicol, nalidixic acid, and ciprofloxacin, while the TEM-153 producer was only resistant to nalidixic acid, ciprofloxacin, and neomycin.

Both ESBLs were successfully transferred by conjugation, and resistance to streptomycin was co-transferred with the *bla*_CTX−M−14_ gene. A clonal relationship was not established between CTX-M-14-producing *E. coli* recovered from healthy volunteers and from Portuguese hospitalized patients or marine coastal waters (Machado et al., 2007, 2009). However, the plasmid containing *bla*_CTX−M−14_, a 80kb IncK-*bla*_CTX−M−14_ plasmid, was similar to that of hospitalized patients from Portugal and other European countries (Valverde et al., 2009; Cottell et al., 2011; data not shown).

### Analysis of integrons

Class 1 and/or class 2 integrons were detected in 9% (19/201) of the isolates, being class 1 integrons more frequently found than class 2 integrons [7% (14/201) *vs.* 3% (6/201)]. Simultaneous presence of class 1 and class 2 integrons was found in one isolate. A low diversity of integrons and of their gene cassettes was observed (Table 2). Five different class 1 integron types were identified, with types II_1_ (*dfrA1-aadA1*, *n* = 3), VI_1_ (*dfrA17-aadA5*, *n* = 3) and XXIV_1_ (*aadA13*, *n* = 3) corresponding to the most prevalent. Among ESBL-producing isolates, class 1 integrons were only identified in the CTX-M-14 producer, with the integron (type II_1_, *dfrA1-aadA1*) and the *bla*_ESBL_ gene being co-transferred by conjugation. Two class 2 integron types were observed: type II_2_ (*dfrA1-sat2-aadA1-orfX*) (*n* = 4) and type III_2_ (*estX-sat2-aadA1-orfX*) (*n* = 1). Gene cassettes were not identified in few isolates harboring *intI1* (2/201, 1%) or *intI2* (1/201, 0.5%).

**Table 2:** **Class 1 and class 2 integron types found among *Enterobacteriaceae* recovered from faecal samples of Portuguese healthy humans**.

**RFLP type**	**Length of variable region (bp)**	**Gene cassettes and order**	**Resistance phenotype^a^**	**No. of isolates**	**Isolation date**
**CLASS 1 INTEGRONS**					
I_1_	1000	*aadA1*	Sm, Sp	2	2001
II_1_	1500	*dfrA1-aadA1*	Tp, Sm, Sp	3^*b^	2003/2004
III_1_	1800	*dfrA12-orfF-aadA2*	Tp, Sm, Sp	1	2001
VI_1_	1500	*dfrA17-aadA5*	Tp, Sm, Sp	3	2001/2003
XXIV_1_	1400	*aadA13*	Sm, Sp	3	2001
**CLASS 2 INTEGRONS**					
II_2_	1900	*dfrA1-sat2-aadA1-orfX*	Tp, Str, Sm, Sp	4	2001/2004
III_2_	2300	*estX-sat2-aadA1-orfX*	Str, Sm, Sp	1	2004

a *Sm, streptomycin; Sp, spectinomycin; Str, streptothricin; Tp, trimethoprim*.

b(*) *One isolate corresponded to the CTX-M-14-producing E. coli*.

### Distribution of *sul* genes

The *sul* genes were found among 88% (22/25) of the sulfonamide resistant isolates, corresponding to *sul1* (48%, 12/25), *sul2* (60%, 15/25), and *sul3* (4%, 1/25), with simultaneous presence of *sul1* and *sul2* in 24% (6/25) of the isolates. A high proportion of *sul* genes was detected among isolates harboring class 1 integrons [64%, 14/22; *sul1*-92% (11/12); *sul2*-47% (7/15); *sul3*-100% (1/1)], corresponding to a prevalence of *sul* genes in integron-positive isolates of 100% (14/14). In one isolate harboring *intI1* but negative for *sul1* we detected the presence of *sul3*.

## Discussion

The rate of faecal carriage of ESBL-producing *Enterobacteriaceae* found in Portuguese healthy persons was similar to that reported in Spain and other countries during the same time period (Valverde et al., 2004; Moubareck et al., 2005; Rodrigues et al., 2005). In Portugal, available data on faecal carriage of ESBL-producing *Enterobacteriaceae* by healthy humans is scarce and restricted to children (Guimarães et al., 2009). However, several works demonstrate a wide dissemination of ESBLs in Portuguese clinical settings, healthy food-producing animals, food products and aquatic environments since 2003, accompanied by a shift in the ESBL-types from TEM- or SHV-types towards CTX-M enzymes (Machado et al., 2004, 2007, 2008, 2009; Mendonça et al., 2007; Peixe and Machado, personal communication). In face of this scenario, the presence of ESBL-producing *Enterobacteriaceae* in the commensal intestinal flora of Portuguese healthy humans was not surprising, being even predictable that future studies will report an increase in the faecal carriage of ESBL producers, as demonstrated in other works (Valverde et al., 2004; Pallecchi et al., 2007; Vinué et al., 2009; Woerther et al., 2010).

The ESBL-producing *E. coli* clones identified belonged to A or B1 phylogenetic groups. These phylogroups have traditionally been considered “commensal” (Johnson and Stell, 2000), although they have been increasingly recovered from human infections, very often from blood cultures (Moreno et al., 2006; Cooke et al., 2010; Rodríguez-Baño et al., 2012; Novais and Peixe, unpublished data). The detection of CTX-M-14-producing *E. coli* isolates from hospitalized patients and marine coastal waters clonally unrelated (phylogroup D) to the one from a Portuguese healthy person in the same time period, but sharing an identical IncK plasmid carrying *bla*_CTX−M−14_ also identified in Spain and other countries (Coque et al., 2008; Valverde et al., 2009; Cottell et al., 2011), highlights the role of transferable plasmids in the global and local spread of antibiotic resistance among different reservoirs. The TEM-153 reported by the first time in this study seems to evolve from *bla*_TEM−1c_ (Leflon-Guibout et al., 2000), a *bla*_TEM−1_ variant identified among animals and predominant among faecal isolates from healthy humans (Briñas et al., 2002). This enzyme is highly similar to TEM-52 (99% homology at aminoacid level, G238S, and V267G), an ESBL-type widely spread in piggeries, chicken meat, and hospitals in Portugal and Europe (Coque et al., 2008; Machado et al., 2008; Peixe and Machado, personal communication).

Although limitations derived from the low number of ESBL-producing isolates included in our study, these data suggest that bacteria colonizing healthy persons constitute a reservoir of new or known ESBL genes that could further evolve at the nosocomial setting and/or be responsible for future epidemic situations. Despite the initial reports of CTX-M-15 producers in several Portuguese hospitals in 2003–2004 (Machado et al., 2007) and the recent studies indicating a high prevalence of CTX-M-15 among the faecal flora of healthy individuals (reflecting its current dominance in the ESBL pandemic) (Moubareck et al., 2005; Geser et al., 2012; Lonchel et al., 2012), this enzyme was not identified during the period analyzed. A later penetration of CTX-M-15 in Portugal (as occurred in other South European countries) (Coque et al., 2008) might have resulted in a lower colonization pressure in these years, in opposite of CTX-M-14 that was already spread in non-nosocomial niches (Machado et al., 2009).

In agreement with previous studies from the same time period (Kang et al., 2005; Skurnik et al., 2005), integrons were found in a low percentage of isolates (9%). All class 1 integron types identified in this study have been extensively found in the hospital and community settings, including Portuguese food-producing animals, with the exception of the integron type XXIV_1_ (containing the gene cassette *aadA13* conferring resistance to streptomycin and spectinomycin) (Machado et al., 2005; Vinué et al., 2008; Ben Sallem et al., 2012). The gene cassette *aadA13* seems to be widespread among Portuguese *E. coli* isolates from pig faeces since 1998 (Machado et al., 2008). Its identification in different species of *Enterobacteriaceae* including firstly in *Morganella morganii*, highlights the highly transmissibility of certain genetic elements carrying antibiotic resistance in this area. Class 2 integrons contents resembled that of Tn*1825* and the widely disseminated Tn*7* (Biskri and Mazel, 2003; Machado et al., 2005).

The occurrence of *sul* genes was similar to that reported in other studies, with *sul2* being more commonly found than *sul1*, and *sul3* being scarcely observed (Enne et al., 2001; Grape et al., 2003; Infante et al., 2005; Hammerum et al., 2006; Trobos et al., 2008; Bailey et al., 2010). These high rates of *sul* genes are identical to those detected among food-producing animals which are highly exposed to sulfonamides, and hence an involvement of the food chain cannot be discarded (Perreten and Boerlin, 2003; Pena et al., 2004; Antunes et al., 2005; Hammerum et al., 2006). The observation of *sul3* in one isolate harboring *intI1* but lacking *sul1* suggests a replacement of *sul1* by *sul3*, as previously reported in similar structures of *Salmonella* and *E. coli* isolates (Antunes et al., 2007; Curião et al., 2011). Although *sul* genes were not observed in some sulfonamide resistant isolates, we could not discard the presence of other resistance mechanisms, as mutations at the chromosomal gene (*folP*) for dihydropteroate synthase (DHPS) (Sköld, 2001) or acquisition of unknown genes.

Prevalence data on intestinal carriage of relevant antibiotic resistance genes and/or structures promoting gene expression in different time periods are important to identify sources and hotspots of antibiotic resistance with relevance for the human health. In summary, data from this retrospective study reinforce the relevance of human commensal flora as reservoir of clinically relevant antibiotic resistance genes (*bla*_CTX−M−14_ or *bla*_TEM153_; *sul*)/genetic platforms (integrons and IncK plasmids). These findings impose future extensive follow-up evaluations in order to understand the trends of the antibiotic resistance genes epidemiology in the community and clinical settings over the time, and eventually anticipate the detection of microorganisms with the potential to cause pandemics in the future.

### Conflict of interest statement

The authors declare that the research was conducted in the absence of any commercial or financial relationships that could be construed as a potential conflict of interest.
